# Pirt promotes substance P release in cancer-induced bone pain

**DOI:** 10.1097/PR9.0000000000001458

**Published:** 2026-06-17

**Authors:** Xueying Cheng, Zhonghua Zhang, Lan Zhou, Jingxiang zeng, Chao Jiang, Shuo Cheng, Yuan Zhou, Guang Yu, Changming Wang

**Affiliations:** a School of Medicine, Nanjing University of Chinese Medicine, Nanjing, Jiangsu, China; b School of Traditional Chinese Medicine, Nanjing University of Chinese Medicine, Nanjing, Jiangsu, China

**Keywords:** Pirt, DRG, Cancer-induced bone pain, Substance P

## Abstract

**Introduction:**

Cancer-induced bone pain (CIBP) is a major clinical challenge due to its intricate, poorly understood pathogenesis. Sensitized tumor-innervating nerves interacting with cancer cells drive CIBP, and substance P (SP) released by these nerves is a key mediator of pathological processes. However, the mechanisms regulating SP release in CIBP remain unclear.

**Objectives:**

To explore Pirt's involvement in CIBP and its role in regulating SP release.

**Methods:**

Molecular and behavioral experiments were performed using Pirt knockout (Pirt-KO) and wild-type (WT) mice in a CIBP model. Pain-related behaviors and SP were assessed, with Pirt expression and localization analyzed in dorsal root ganglia (DRG) and spinal cord.

**Results:**

Pirt-KO mice exhibited attenuated heat allodynia, mechanical allodynia, and spontaneous pain vs WT mice. Substance P level in tissues and SP release from DRG neurons was significantly reduced in Pirt-KO mice. All DRG SP-positive neurons expressed Pirt, and Pirt – green fluorescent protein (GFP) terminals projected to spinal lamina I/II, colocalizing with SP-positive terminals.

**Conclusion:**

Pirt plays a crucial role in promoting SP release in CIBP, shedding light on the molecular mechanisms underlying CIBP and providing a potential therapeutic target.

## Abstract 2

Supplemental Digital Content is Available in the Text.

Pirt promotes substance P release to exacerbate cancer-induced bone pain, emerging as a potential therapeutic target.

## 1. Introduction

Cancer-induced bone pain (CIBP) constitutes the most debilitating complication for patients with cancer with osteosarcoma or bone metastases. More than 80% of patients with metastatic cancer experience CIBP, which significantly diminishes their overall quality of life.^23^ Despite its prevalence, the underlying mechanisms of CIBP are not yet fully understood, with sensitized tumor-innervating sensory nerves and their bidirectional crosstalk with cancer cells emerging as core drivers of persistent pain signaling.

Substance P (SP), a neuropeptide consisting of 11 amino acids, is released from the peripheral terminals of activated sensory nerve fibers on stimulation and has been demonstrated to play a critical role in modulating pain sensation.^5,10,11^ It modulates nociceptive transmission and, as we recently reported, drives macrophage migration in the primary phase of CIBP.^17^ However, the upstream molecular mechanisms governing SP release in CIBP remain largely undefined, representing a critical gap in targeted therapeutic development.

Pirt, a 2-transmembrane phosphoinositide-binding protein specifically expressed in dorsal root ganglia (DRG) neurons, is a conserved positive regulator of nociceptive pain transduction, with its mechanistic regulation of the transient receptor potential vanilloid 1 (TRPV1) channel being its most well-characterized function.^9^ Pirt-dependent modulation of TRPV1 is indispensable for nociceptive signaling: Pirt knockout (Pirt-KO) mice exhibit impaired heat pain responses in neuropathic pain models,^15^ and the Pirt-TRPV1 axis is also implicated in uterine contraction-induced pain^19^ and neuropathic pain.^10,13^ TRPV1 is a well-established mediator of CIBP,^3,4^ establishing a potential functional link between Pirt and SP release in CIBP.

While Pirt is known to regulate pain sensation in multiple pathological states, its specific role in CIBP remains largely unexplored. Here, we aimed to investigate the effects of Pirt regulation in CIBP using Pirt-KO and wild-type (WT) mice. Through behavioral assessments of pain hypersensitivity and molecular quantification of SP release and expression, we determined that Pirt significantly promotes SP expression and release in CIBP, uncovering a novel molecular mechanism underlying this clinically intractable pain and identifying a potential therapeutic target.

## 2. Material and methods

### 2.1. Animals

Animal experiments (approved by Nanjing Univ. of Chinese Med. Ethics Committee: ACU190601; IASP guidelines) used 8-week-old (20–25 g) WT, Pirt-KO (Johns Hopkins lab). Mice were housed SPF (specific pathoen-free), weaned at 3 weeks, with free food/water; experiments analyzed by genotype-blinded researchers. Detailed experimental protocols are provided in the Supplementary Materials, http://links.lww.com/PR9/A416.

### 2.2. Cancer-induced bone pain model

The CIBP model was performed as previously described.^7,8,17^

### 2.3. Hargreaves test

Hargreaves tests were performed using littermate control mice, following previously established protocols.^7,12,18^

### 2.4. Von Frey test

Von Frey tests were conducted using littermate control mice, as described in previous studies.^16^

### 2.5. Real-time polymerase chain reaction (PCR)

The protocols and materials are the same as we did before.^6,19^ Primers for Tac1-SP forward primer: CCG​ACA​GTG​ACC​AGA​TCA​AGG; reverse primer: GCA​TCC​CGC​TTG​CCC​ATT​A. Primers for GADPH forward primer: ACC​ACA​GTC​CAT​GCC​ATC​AC; reverse primer: TCC​ACC​ACC​CTG​TTG​CTG​TA.

### 2.6. Immunofluorescence staining of dorsal root ganglia

Immunostaining was performed as we reported previously.^14,16^ Primary antibody of SP: Anti-Substance P antibody (Abcam, ab7340, 1:200, Shanghai, China). Primary antibody of NeuN: Anti-NeuN antibody Neuronal Marker (Abcam, ab236870, 1:200).

### 2.7. ELISA

ELISA (mlbio, ml001885) quantified DRG substance P release per manufacturer's protocol as previously shown.^17^

### 2.8. Statistical analysis

Data are mean ± SD. Normal-distributed data were analyzed by unpaired *t* test/ANOVA (SPSS 16.0) with post hoc tests; *P* < 0.05 was significant.

## 3. Results

### 3.1. Pirt promotes cancer-induced bone pain–induced-heat, mechanical allodynia, and spontaneous pain

**Figure 1.**
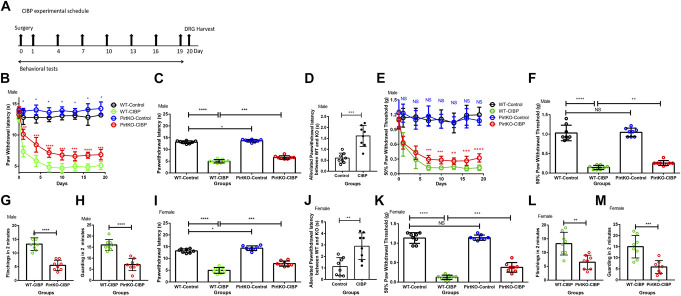
Pirt promotes heat, mechanical allodynia, and spontaneous pain in cancer-induced bone pain. (A) Experimental schedule in CIBP model. (B) The heat allodynia (Paw withdrawal latency) was recorded at 1, 4, 7, 10, 13, 16, and 19 days post-CIBP surgery in WT and Pirt-KO male mice. Statistical significance markers: blue * indicates comparison between Baseline WT and Baseline Pirt-KO groups; red * indicates comparison between WT-CIBP and Pirt-KO-CIBP groups at the corresponding time point. (C) The heat allodynia of WT control group (heat-inactivated Lewis lung cancer cells), WT CIBP mice, Pirt control group (heat-inactivated Lewis lung cancer cells), and Pirt-KO CIBP group were compared at 13 days post-CIBP surgery, n = 8 mice/group. (D) Quantification of Pirt-enhanced heat allodynia (calculated as the difference in Paw withdrawal latency between control and CIBP) in WT and Pirt-KO male mice. (E) The mechanical allodynia (50% paw withdrawal threshold) was recorded at 1, 4, 7, 10, 13, 16, and 19 days post-CIBP surgery in WT and Pirt-KO male mice. Statistical significance markers: blue NS indicates comparison between Baseline WT and Baseline Pirt-KO groups; red * indicates comparison between WT-CIBP and Pirt-KO-CIBP groups at the corresponding time point. (F) The mechanical allodynia of WT control group, WT CIBP mice, Pirt control group, and Pirt-KO CIBP group were compared at 13 days post-CIBP surgery, n = 8 mice/group. (G and H) The flinchings (G) and guardings (H) of WT and Pirt-KO male mice were compared in CIBP model at 13 days post-CIBP surgery, n = 8 mice/group. (I) The mechanical allodynia of WT control group, WT CIBP group, Pirt control group, and Pirt-KO CIBP group in female mice were compared at 13 days post-CIBP surgery, n = 8 mice/group. (J) Quantification of Pirt-enhanced heat allodynia (calculated as the difference in Paw withdrawal latency between control and CIBP) in WT and Pirt-KO female mice. (K) The heat allodynia of WT control group, WT CIBP group, Pirt control group and Pirt-KO CIBP group in female mice were compared at 13 days post-CIBP surgery, n = 8 mice/group. (L and M) The flinchings (L) and guardings (M) of WT and Pirt-KO female mice were compared in CIBP model at 13 days post-CIBP surgery, n = 8 mice/group. NS, no significant difference, *P < 0.05, **P < 0.01, ***P < 0.001, ****P < 0.0001. Paired (D, G, H, J, L, M) *t* test. Two-way ANOVA (B and E) and one-way ANOVA (C, F, I, K) followed by the Bonferroni post hoc test; error bar, SD. CIBP, cancer-induced bone pain; KO, knockout; WT, wild-type.

To examine the role of Pirt in CIBP, we used a murine model. Heat and mechanical allodynia were evaluated on days 1, 4, 7, 10, 13, 16, and 19 days post-CIBP surgery (Fig. 1A). Our findings demonstrated an increase in heat allodynia in the CIBP model relative to the control group. Notably, this increase was significantly attenuated in Pirt-deficient mice (Pirt-KO mice) (Fig. 1B, C, D). In addition, the heightened mechanical allodynia observed in the CIBP model was also mitigated in Pirt-KO mice compared with WT mice (Fig. 1E, F). Spontaneous pain (flinching and guarding behaviors) was specifically evaluated at 13 days postsurgery; in Pirt-KO mice, both flinching (Fig. 1G) and guarding behaviors (Fig. 1H) were significantly reduced compared with WT mice in CIBP. For female mice, mechanical and heat allodynia were assessed at 13 days post-CIBP surgery (Fig. 1I, J, K), and spontaneous pain (flinching and guarding) was also evaluated at this time point (Fig. 1L, M). No spontaneous pain was detected in either WT or Pirt-KO mice within the control group; however, in the CIBP model, spontaneous pain was reduced in Pirt-KO mice compared with WT mice (Fig. 1L, M). Thus, Pirt promotes the heat allodynia, mechanical allodynia, and spontaneous pain in cancer-induced bone pain.

### 3.2. Pirt promotes the release of substance P in physiological conditions and cancer-induced bone pain

**Figure 2.**
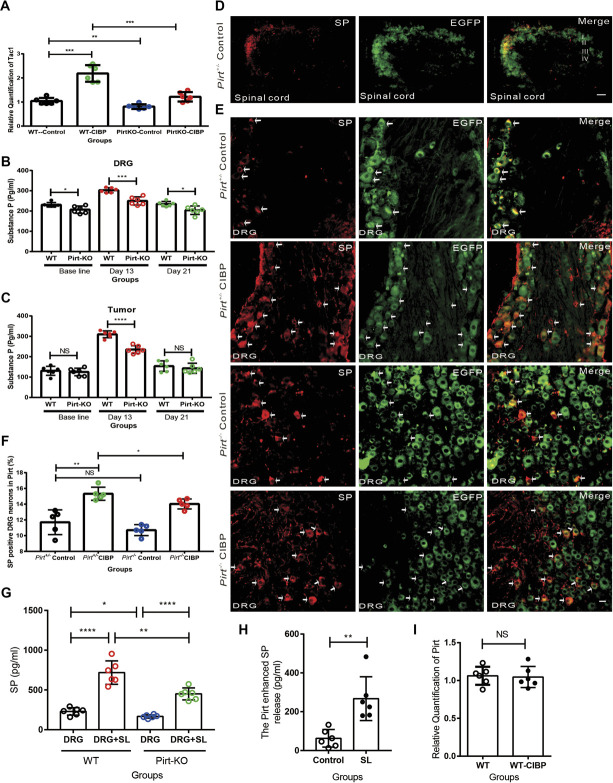
Substance P expression is alleviated in Pirt-KO mice in CIBP model. (A) Real-time PCR results showed the transcriptional expression of Tac1 in L4 and L5 DRGs in WT control group (heat-inactivated Lewis lung cancer cells), WT CIBP group, Pirt-KO control group (heat-inactivated Lewis lung cancer cells), Pirt-KO CIBP group at 13 days post-CIBP surgery (n = 6 mice/group). (B) ELISA results showed the release of SP in L4 and L5 DRGs in WT CIBP group and Pirt-KO CIBP group on Control (heat-inactivated Lewis lung cancer cells), 13th day and 21st day (n = 6 mice/group). (C) ELISA results showed the release of SP in tumor tissue in WT CIBP group and Pirt-KO CIBP group at baseline, 13 days, and 21 days post-CIBP surgery (n = 6 mice/group). (D) Representative pictures showed SP expression and its colocalization with Pirt in *Pirt*^*+/-*^ mice in spinal cord at 13 days post-CIBP surgery. Scale Bar: 50 μm. (E) Representative pictures showed SP expression and its colocalization with Pirt in DRG neurons in *Pirt*^*+/-*^ control group, *Pirt*^*+/-*^ CIBP group, *Pirt*^*-/-*^ control group, and *Pirt*^*-/-*^ CIBP group on 13th day. Scale Bar: 20 μm. (F) Percentages of SP positive neurons in DRGs of *Pirt*^*+/-*^ control group, *Pirt*^*+/-*^ CIBP group, *Pirt*^*-/-*^ control group, and *Pirt*^*-/-*^ CIBP group were quantified at 13 days post-CIBP surgery (n = 5 mice/group). (G) SP concentration in supernatant (pg/mL) in cultured DRG neurons from wild-type (WT) and Pirt-knockout (Pirt-KO) mice, with or without 12-hour incubation with SL (supernatant liquid of Lewis lung cancer cells). (H) Quantification of SL-enhanced SP release (calculated as the difference in SP levels between SL-treated and untreated DRG neurons) in WT and Pirt-KO mice. (I) mRNA expression of Pirt in DRGs in CIBP mice. NS, no significant difference, *P < 0.05, **P < 0.01, ***P < 0.001. One-way ANOVA (A, B, C, F, G) followed by the Bonferroni's post hoc test, paired (H) and unpaired *t* test (I); error bar, SD. CIBP, cancer-induced bone pain; DRG, dorsal root ganglia; KO, knockout; SP, Substance P; WT, wild-type.

Substance P release is critical in pain sensation, especially in CIBP.^17^ We investigated the expression of *Tac-1* mRNA (encoding the precursor of substance P) in L4/L5 DRG neurons of Pirt-KO and WT mice at 13 days post-CIBP surgery. Unexpectedly, our findings revealed a reduction in *Tac-1* mRNA level in the DRG of Pirt-KO mice under homeostatic conditions, a phenomenon not previously reported (Fig. 2A). Furthermore, the *Tac-1* mRNA levels were significantly lower in Pirt-KO mice compared with WT mice within the CIBP model (Fig. 2A). ELISA was used to detect SP level in L4/L5 DRGs at baseline, 13 days, and 21 days post-CIBP surgery; in both control and CIBP mice, SP levels were consistently reduced in Pirt-KO mice at all these time points (Fig. 2B). These results suggest that Pirt enhances SP expression under both physiological and pathological conditions.

In addition, SP levels in tumor tissues were detected through ELISA at baseline, 13 days, and 21 days post-CIBP surgery; SP was elevated in WT mice within the CIBP model, while this increase was mitigated in Pirt-KO CIBP mice (Fig. 2C). Furthermore, we confirmed the colocalization of SP and Pirt through immunofluorescence staining in *Pirt*^*+/-*^ mice at 13 days post-CIBP surgery. Pirt-GFP terminals were colocalized with SP-positive terminals in the spinal cord, projecting to lamina I and lamina II (Fig. 2D). Notably, fluorescence density of SP-positive terminals was lower than that of Pirt-GFP terminals. Similarly, in the DRG, SP-positive neurons were also colocalized with Pirt-GFP neurons at 13 days post-CIBP surgery (Fig. 2E). Subsequent experiments demonstrated an increase in the number of SP-positive DRG neurons in CIBP mice at 13 days postsurgery, which was attenuated in Pirt-KO mice (Fig. 2E, F). To further clarify Pirt promotes SP release in CIBP, we measured SP release from dissociated DRG neurons of WT and Pirt-KO mice. Consistent with our prior findings, supernatant liquid of Lewis lung cancer cells (SL) stimulation induced a marked increase in SP release from WT DRG neurons (Fig. 2G). Notably, Pirt-KO DRG neurons exhibited a significant reduction in basal SP release, and this reduction was further amplified in SL-stimulated conditions (Fig. 2G, H), providing direct, definitive experimental evidence that Pirt is essential for promoting SP release in CIBP. However, the expression of Pirt in DRGs, as measured by real-time PCR, remained unchanged (Fig. 2I); Overall, these results confirmed the role of Pirt in promoting SP releases in CIBP.

## 4. Discussion

In this study, we found a novel mechanism that contributes to CIBP, demonstrating that Pirt promotes SP release in mice afflicted with CIBP.

Notably, we observed a significant reduction in both thermal and mechanical allodynia in Pirt-KO mice with CIBP. Our previous research indicated that Pirt-KO mice exhibit impaired responses to heat stimuli in neuropathic pain.^15^ This study identified a similar trend in CIBP, with an even greater disparity observed. Conversely, no significant difference in mechanical sensitivity was detected between the WT and Pirt-KO control mice, aligning with our previous findings.^15^ However, a distinct difference was observed between WT and Pirt-KO mice in the CIBP model. Furthermore, spontaneous pain was mitigated in Pirt-KO mice after assessment in the CIBP model. In this study, we found that Pirt contributes to CIBP and regulates SP release in both male and female mice, with no obvious sex differences observed in the effect of Pirt on CIBP-related behaviors. These results suggest that Pirt exacerbates heat allodynia, mechanical allodynia, and spontaneous pain in CIBP in male and female mice without sex differences.

The interactions between nerve and tumor cells are of considerable importance in the mechanisms underlying CIBP.^20,21^ Substance P, a member of the neuropeptide family, is released from nerve endings and plays a critical role in cancer progression.^1^ Our recent study further proposed a 2-phase CIBP model: SP-NK1R mediates early macrophage recruitment, while CCL2/CCL3 sustains late pain.^17^ We further validated THP as a potential therapy, which inhibits TRPV1-SP signaling and promotes M2 macrophage polarization, aligning with the 2-phase mechanism and providing a translational strategy for CIBP treatment.^22^ In this study, we identified SP-positive DRG neurons and observed that SP tissue level peaked on the 13th day after CIBP induction. Our subsequent investigations revealed the colocalization of Pirt and SP in laminae I and II of the spinal cord, regions where small-diameter neurons terminate. Furthermore, both Pirt and SP were coexpressed in small-diameter DRG neurons, with Pirt also being expressed in medium-diameter DRG neurons.^9^ These colocalization patterns suggest a close relationship between Pirt and SP. Our study identified a significant reduction in the expression of SP in Pirt-KO mice, with this reduction being further pronounced in CIBP. Moreover, Pirt deletion reduced basal SP release in DRG neurons, and the difference was further enlarged under SL stimulation, directly confirming Pirt is essential for SP release in CIBP. This finding suggests that Pirt promotes SP release under both physiological and CIBP conditions.

In addition, our analysis of the DRGs revealed that Pirt expression remains unchanged. Extensive researches indicate that TRPV1 is implicated in CIBP.^2,21^ TRPV1 is a nonselective cation channel regulated by Pirt.^9,19^ These researches prompted the hypothesis that Pirt modulates TRPV1 activity in CIBP.

In this study, our findings offer compelling evidence for the role of Pirt in promoting the release of substance P in DRG neurons, presenting a novel mechanism underlying the manifestation of cancer-induced bone pain.

## Disclosures

The authors have no conflict of interest to declare.

## Supplemental digital content

Supplemental digital content associated with this article can be found online at http://links.lww.com/PR9/A416.
