# Supplementary material for: Pirt promotes substance P release in cancer-induced bone pain
Source: Pain Rep. 2026 Jun 17;11(4):e1458. doi: 10.1097/PR9.0000000000001458 (PMC13278241; doi:10.1097/PR9.0000000000001458)
Supplement: Supplementary file 1 [file painreports-11-e1458-s001.pdf]

1. Genotyping of Pirt-KO mice:

Genomic DNA Extraction Genomic DNA was isolated from mouse tail snips using a standard lysis and ethanol precipitation method. Briefly, tissue samples were digested overnight at 55 °C in 500 μL of lysis buffer (100 mM Tris-HCl, 5 mM EDTA, 200 mM NaCl, 0.2% SDS) supplemented with Proteinase K (Solarbio, Beijing, China; Cat# P9460). The lysate was centrifuged at 5,000 rpm for 5 min, and 200 μL of the supernatant was collected. DNA was precipitated by the addition of 400 μL absolute ethanol, followed by centrifugation at 12,000 rpm for 5 min. The DNA pellet was washed with 70% ethanol, air-dried for at least 30 min, and dissolved in 30 μL of TE buffer.

PCR Amplification and Electrophoresis Genotyping of the Pirt allele was performed via polymerase chain reaction (PCR). The PCR reaction mixture (25.5 μL total volume) consisted of 12.5 μL of Taq PCR Master Mix (Yeasen, Shanghai, China; Cat# 10102ES03), 2.5 μL of genomic DNA template, and specific primers (Pirt-F: CAACTTTGTGGTACCCGAAG; Pirt-R: TCCCTGGGACTCATGATGCT). The PCR cycling conditions were as follows: initial denaturation at 95°C for 5 min; followed by 38 cycles of 94°C for 30 s, 62°C for 30 s, and 72°C for 30 s; with a final extension at 72°C for 10 min. Amplified PCR products were separated by electrophoresis on a 2% (w/v) agarose gel (Solarbio, Beijing, China; Cat# A8201) in TAE buffer at 120 V for 30 minutes. The expected amplicon sizes were 637 bp for the wild-type (WT) allele and 450 bp for the mutant allele.

2. Pirt knockout (*Pirt*$^{-/-}$) mice and *Pirt*$^{+/-}$ heterozygous mice

Pirt knockout (*Pirt*$^{-/-}$) mice and *Pirt*$^{+/-}$ heterozygous mice (used for Pirt expression visualization) were kindly provided by the laboratory of Dr. Xinzhong Dong (Johns Hopkins University). The *Pirt*$^{-/-}$ null allele was generated by homologous recombination, in which the entire endogenous Pirt coding region was replaced with a targeting construct containing EGFP-IRES-rtTA-ACN[4; 6; 9]. In this knock-in system, EGFP is expressed under the control of the endogenous Pirt promoter, thus serving as a specific reporter for the spatiotemporal expression pattern of Pirt. *Pirt*$^{+/-}$ heterozygous mice (retaining one functional Pirt allele and one EGFP knock-in allele) were used for

immunofluorescence colocalization analysis of Pirt and SP (Figure 2D-E), as EGFP fluorescence in these mice directly labels Pirt-expressing neurons/terminals. *Pirt*$^{-/-}$ homozygous and *Pirt*$^{+/+}$ wild-type littermates were generated by breeding Pirt$^{+/-}$ heterozygotes and used for behavioral and molecular experiments. All mice were 8 weeks old (20-25 g) at the start of experiments, housed in SPF conditions, weaned at 3 weeks, with free access to food and water.

3. Additional ARRIVE E10-aligned details

3.1. Standardization of behavioral test procedures (same time of day, same room, experimenter details)

All pain-related behavioral assessments (Hargreaves test for heat allodynia, Von Frey test for mechanical allodynia, and spontaneous pain scoring for flinching/guarding behaviors) were performed in the same temperature- and humidity-controlled behavioral testing room (22±2°C, 50±5% humidity) with a 12 h light/dark cycle (lights on 08:00-20:00) as we did previously[2; 7]. All tests were conducted by two trained, genotype-blinded experimenters (Xueying Cheng and Zhonghua Zhang) at the same time window daily (10:00-14:00) to eliminate circadian and inter-experimenter variability. Mice were acclimatized to the testing room for at least 30 min before each behavioral assay to reduce stress-induced confounding factors.

3.2. Total number of animals used in the study

A total of 134 specific pathogen-free (SPF) 8-week-old C57BL/6 and Pirt-KO mice (20-25 g) were used in experiments:

Behavioral tests (Hargreaves, Von Frey, spontaneous pain): n = 8 mice per group (WT-Control, WT-CIBP, Pirt-KO-Control, Pirt-KO-CIBP) for both male and female cohorts (64 mice total).

Only male mice were used in molecular experiments (qPCR, ELISA, immunofluorescence): n = 6 mice per group for qPCR/ELISA (24 +36 =50 mice) and n = 5 mice per group for immunofluorescence (20 mice. The mice in behavioral tests were not used for molecular experiments as the molecular experiments were on 13th day after the CIBP surgery).

3.3. Animal exclusion criteria and exclusion status

No animals were excluded from the final data analysis, for the following reasons:

All mice were healthy with no pre-existing neurological or musculoskeletal abnormalities before experimental induction, as confirmed by pre-study physical and behavioral screening.

No mice died during the CIBP model establishment or experimental period (19 days post-surgery).

All mice completed the full behavioral test and tissue collection protocols; no samples were lost or contaminated during molecular experiments (qPCR, ELISA, immunofluorescence).

3.4. A priori sample size calculation

A priori sample size calculation was performed using G*Power 3.1 software (Heinrich-Heine-Universität Düsseldorf, Germany) to determine the minimum number of mice required to detect a statistically significant effect, based on our previous published data on Pirt-mediated nociceptive pain and CIBP models [2; 3; 6; 7; 9].

4. The details of CIBP surgery

Mice were anesthetized via intraperitoneal injection of 2% pentobarbital sodium at a dose of 50 mg/kg (Merck, Cat. No. P11011), consistent with the protocols established in our previous studies [5; 7; 11]. Following anesthesia, mice were placed in a supine position; the hair over the right knee joint was shaved, and the surgical site was thoroughly disinfected with 75% ethanol to maintain aseptic conditions. The hind limb was fixed in a neutral position to expose the knee joint, and a longitudinal skin incision of approximately 0.5 cm was made over the joint capsule. The capsule was then carefully incised to expose the patellar ligament, which was transected to gain clear access to the femoral intercondylar fossa (this transection step was omitted in all control/sham groups). A small burr hole was drilled into the femoral intercondylar fossa along the long axis of the femur using a sterile electric drill, and 10 μL of Lewis lung cancer cell suspension (TCM7 cell line, C57BL/6 genetic background, 1×10^6 cells; Cell Bank of the Chinese Academy of Sciences) was slowly and carefully injected into the

femoral medullary cavity using a microsyringe to avoid cell extravasation. For the control group, an equal volume of heat-inactivated TCM7 cells (incubated at 50℃ for 40 min to ensure complete cell inactivation) was injected using the identical surgical procedure. Immediately after injection, the drill hole was sealed with sterile bone wax and monitored for 2 min to confirm no leakage of the cell suspension. The joint capsule and skin incision were then sutured layer by layer with sterile surgical suture; the surgical site was re-disinfected with 75% ethanol and topically treated with antibacterial ointment to prevent infection. Post-surgery, mice were placed individually in a clean, warm cage and closely observed until full recovery from anesthesia, with regular monitoring of postoperative health and wound healing thereafter.

5. The details of behavioral tests

5.1 Hargreaves Test (Heat Allodynia)

Heat allodynia was assessed using the Hargreaves test (Ugo Basile, Gemonio, Italy) as previously described [8,20]. Mice were acclimated in a transparent Plexiglas chamber (20×10×15 cm) on a glass plate (30 ± 0.5 ℃) for 30 min before testing to eliminate stress-induced behavioral bias. A focused infrared heat source was applied to the plantar surface of the hind paw (ipsilateral to the tumor-injected femur), and the paw withdrawal latency (PWL) (s) was recorded as the primary endpoint for heat allodynia. A cut-off latency of 20 s was set to prevent tissue damage to the paw. Each mouse was tested 3 times at 5-min intervals, and the average value was used for statistical analysis. Heat allodynia was evaluated on days 1, 4, 7, 10, 13, 16, and 19 post-surgery.

5.2 Von Frey Test (Mechanical Allodynia)

Mechanical allodynia was measured using the up-down Von Frey filament test (0.04 to 2 g, Ugo Basile, Gemonio, Italy) as previously described [7; 9] with minor modifications. Mice were acclimated in a wire-mesh chamber (20×10 ×15 cm) for 30 min before testing. A series of calibrated Von Frey filaments were applied vertically to the plantar surface of the ipsilateral hind paw for 3-5 s with sufficient force to cause slight bending of the filament. Positive reactions (foot shrinkage, licking, bouncing) were marked “X,” negatives “O,” with 3 min intervals. The 50% mechanical retraction

threshold was determined by the "UP-and-Down" method as before [7; 8].

5.3 Spontaneous Pain Assessment

Spontaneous pain-related behaviors (flinching and guarding) were evaluated as previously described [1; 10] on day 13 post-surgery (the peak of spontaneous pain in our CIBP model). Mice were placed in a clean Plexiglas chamber (20×20×15 cm) with a white paper floor and acclimated for 15 min. After acclimation, the mouse's behavior was video-recorded for 2 min by a genotype-blinded observer, and the following spontaneous pain endpoints were quantified:

Flinching: The number of rapid, involuntary contractions of the ipsilateral hind paw within 2 min;

Guarding: The total duration (s) of the ipsilateral hind paw being lifted or held close to the body (excluding normal locomotion) within 2 min.

No spontaneous pain behaviors were observed in the control group (heat-inactivated Lewis lung cancer cell injection), and only the CIBP group (WT and Pirt-KO) was subjected to spontaneous pain quantification, with the same assessment protocol applied to both male and female mice.

References:

[1] Ding Z, Liang X, Wang J, Song Z, Guo Q, Schäfer MKE, Huang C. Inhibition of spinal ferroptosis-like cell death alleviates hyperalgesia and spontaneous pain in a mouse model of bone cancer pain. Redox Biology 2023;62.

[2] Ji H, Jin X, Zhang Q, Zhou Y, Zhu C, Yang Y, Tang Z, Yu G, Wang C. A Mouse Model of Cancer Induced Bone Pain: From Pain to Movement. Front Behav Neurosci 2022;16:873750.

[3] Jin X, Cheng J, Zhang Q, Ji H, Zhu C, Yang Y, Zhou Y, Yu G, Wang C, Tang Z. Aconitine – A promising candidate for treating cold and mechanical allodynia in cancer induced bone pain. Biomedicine & Pharmacotherapy 2023;161(114284).

[4] Kim AY, Tang Z, Liu Q, Patel KN, Maag D, Geng Y, Dong X. Pirt, a phosphoinositide-binding protein, functions as a regulatory subunit of TRPV1. Cell 2008;133(3):475-485.

[5] Ruan Y, Gu L, Yan J, Guo J, Geng X, Shi H, Yu G, Zhu C, Yang Y, Zhou Y, Wang C, Tang Z. An effective and concise device for detecting cold allodynia in mice. Sci Rep 2018;8(1):14002.

[6] Wang C, Gu L, Ruan Y, Gegen T, Yu L, Zhu C, Yang Y, Zhou Y, Yu G, Tang Z. Pirt Together with TRPV1 Is Involved in the Regulation of Neuropathic Pain. Neural Plast 2018;2018:4861491.

[7] Wang C, Ji H, Wang H, Chen Z, Zhou L, Yang Y, Jiang Y, Yu G, Jiang L, Tang Z. Unraveling the neuroimmune mechanisms in cancer-induced bone pain: New horizons for therapeutic

intervention of the two-phase paradigm. Proc Natl Acad Sci U S A 2025;122(34):e2503779122.

[8] Wang C, Jin X, Zhang Q, Wang H, Ji H, Zhou Y, Zhu C, Yang Y, Yu G, Tang Z. TRPV1 and TRPA1 channels interact to mediate cold hyperalgesia in mice. British Journal of Anaesthesia 2023;131(5):4.

[9] Wang C, Wang Z, Yang Y, Zhu C, Wu G, Yu G, Jian T, Yang N, Shi H, Tang M, He Q, Lan L, Liu Q, Guan Y, Dong X, Duan J, Tang Z. Pirt contributes to uterine contraction-induced pain in mice. Mol Pain 2015;11:57.

[10] Wang K, Donnelly CR, Jiang C, Liao Y, Luo X, Tao X, Bang S, McGinnis A, Lee M, Hilton MJ, Ji RR. STING suppresses bone cancer pain via immune and neuronal modulation. Nat Commun 2021;12(1):4558.

[11] Zhang Q, Chen Z, Yu Q, Wang H, Jiang Y, Zhou L, Yu G, Tang Z, Wang C. Tetrahydropalmatine alleviates cancer induced bone pain by inhibiting TRPV1-SP-mediated macrophage recruitment and promoting M2 polarization. Chinese Medicine 2026;21(1).
